# Endometriosis: clinical features, MR imaging findings and pathologic correlation

**DOI:** 10.1007/s13244-017-0591-0

**Published:** 2018-02-15

**Authors:** Pietro Valerio Foti, Renato Farina, Stefano Palmucci, Ilenia Anna Agata Vizzini, Norma Libertini, Maria Coronella, Saveria Spadola, Rosario Caltabiano, Marco Iraci, Antonio Basile, Pietro Milone, Antonio Cianci, Giovanni Carlo Ettorre

**Affiliations:** 1 Radiodiagnostic and Radiotherapy Unit, University Hospital “Policlinico-Vittorio Emanuele”, Via Santa Sofia 78, 95123 Catania, Italy; 2 Department G.F. Ingrassia – Institute of Pathology, University of Catania, Catania, Italy; 3 Department of General Surgery and Medical-Surgical Specialties – Institute of Obstetrics and Ginecology, University of Catania, Catania, Italy

**Keywords:** Endometriosis, Magnetic resonance imaging, Endometrioma, Deep infiltrating endometriosis, Pelvis, Pelvic pain

## Abstract

**Objective:**

We illustrate the magnetic resonance imaging (MRI) features of endometriosis.

**Background:**

Endometriosis is a chronic gynaecological condition affecting women of reproductive age and may cause pelvic pain and infertility. It is characterized by the growth of functional ectopic endometrial glands and stroma outside the uterus and includes three different manifestations: ovarian endometriomas, peritoneal implants, deep pelvic endometriosis. The primary locations are in the pelvis; extrapelvic endometriosis may rarely occur. Diagnosis requires a combination of clinical history, invasive and non-invasive techniques. The definitive diagnosis is based on laparoscopy with histological confirmation. Diagnostic imaging is necessary for treatment planning. MRI is as a second-line technique after ultrasound. The MRI appearance of endometriotic lesions is variable and depends on the quantity and age of haemorrhage, the amount of endometrial cells, stroma, smooth muscle proliferation and fibrosis. The purpose of surgery is to achieve complete resection of all endometriotic lesions in the same operation.

**Conclusion:**

Owing to the possibility to perform a complete assessment of all pelvic compartments at one time, MRI represents the best imaging technique for preoperative staging of endometriosis, in order to choose the more appropriate surgical approach and to plan a multidisciplinary team work.

**Teaching Points:**

*• Endometriosis includes ovarian endometriomas, peritoneal implants and deep pelvic endometriosis.*

*• MRI is a second-line imaging technique after US.*

*• Deep pelvic endometriosis is associated with chronic pelvic pain and infertility.*

*• Endometriosis is characterized by considerable diagnostic delay.*

*• MRI is the best imaging technique for preoperative staging of endometriosis.*

## Introduction

Endometriosis is a chronic multifocal gynecologic disease that affects women of reproductive age and may cause pelvic pain and infertility. The aetiology of endometriosis is unknown [1]; the pathogenesis is complex, multifactorial and still debated. The disease is characterized by the growth of functional ectopic endometrial glands and stroma outside the uterus [1–3]. Its prevalence is of approximately 10% in women of reproductive age, 20–50% in women with infertility and nearly 90% in women with chronic pelvic pain [1, 3]. It is thought that the disease affects about 176 million women of reproductive age worldwide [4], with a peak of incidence between 24 and 29 years [5].

The disease includes three different manifestations, namely ovarian endometriomas, superficial peritoneal implants, and deep pelvic endometriosis. The latter is defined as endometriotic lesions penetrating into the retroperitoneal space or the wall of the pelvic organs to a depth of at least 5 mm; it has an estimated prevalence of 1% among women of reproductive age [6] and affects up to 20% of women with endometriosis [7].

Although the definitive diagnosis is based on laparoscopy or surgery with histological verification of endometrial glands and/or stroma, imaging is necessary for treatment planning.

Among imaging modalities, magnetic resonance imaging (MRI) is often used as a problem-solving additional examination in complex cases and should be considered as a second-line technique after ultrasound (US) [1]. Currently, MRI is considered the best imaging technique for mapping endometriosis, since it provides a more reliable map of deep infiltrating endometriosis than physical examination and transvaginal ultrasound (TVUS) [8].

In this article we review clinical manifestations and histological features of endometriosis. We describe MR imaging appearances of the different manifestations of pelvic endometriosis and some extrapelvic locations, using examples, many of which pathologically proven, from our institution. Our MR protocol is also included.

## Locations and clinical features

**Fig. 1:**
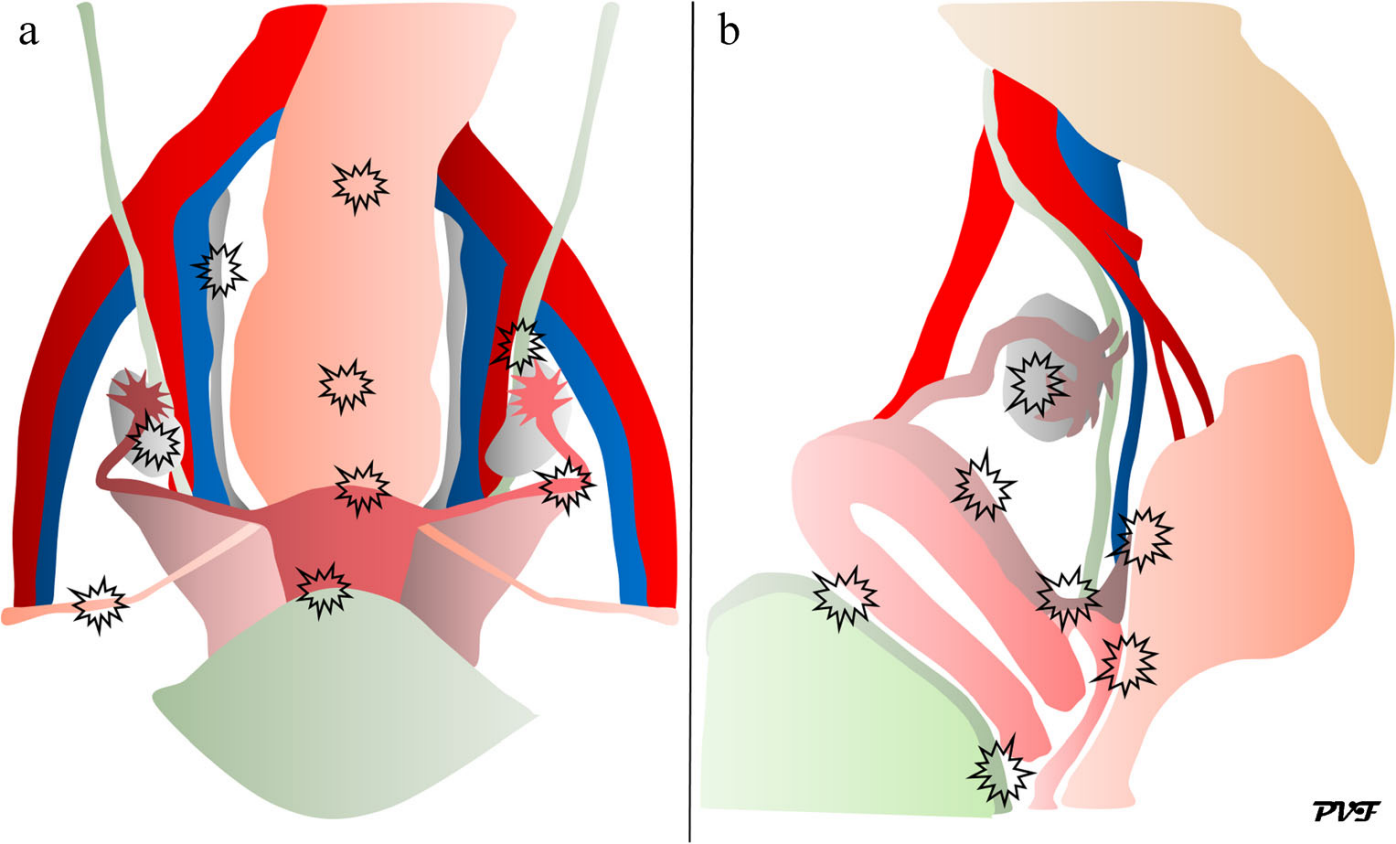
Drawings of the female pelvis in the **(a)** ventral and **(b)** lateral views illustrate the primary locations of endometriotic lesions

The primary locations of endometriosis are in the pelvis: on the ovaries, uterus, fallopian tubes, uterosacral ligaments (USL), broad ligaments, round ligaments, cul-de-sac, rectosigmoid colon, bladder, ureters, and rectovaginal septum (RVS) (Fig. 1) [9]. Endometriotic locations with corresponding prevalence are listed in Table 1 [3, 10, 11].

**Table 1:** Primary locations of endometriosis, their prevalence in patients with endometriosis, clinical features and differential diagnosis [3, 10, 11]

Locations	%	Clinical features	MR differential diagnosis
Bladder	6.4–20	dysuria, hematuria, urinary storage symptoms, suprapubic pain	urachal remnant, epithelial and mesenchymal tumours
Ureters	0.01–1	dysmenorrhea, dyspareunia, flank pain (hydronephrosis)	obstruction by cervical cancer
Ovaries	20–40	nonspecific pelvic pain	dermoids, hemorrhagic cysts, endometrioid and clear cell tumours
Round ligaments	0.3–14	painful inguinal mass, nonspecific pelvic pain	
Retrocervical region, uterosacral ligaments	69.2	painful symptoms, dyspareunia	peritoneal metastases
Vagina	14.5	dysmenorrhea, dyspareunia, postcoital spotting	cervical and vaginal carcinoma
Rectosigmoid colon	9.9–37	dyschezia, cyclic pain, rectal bleeding	colorectal cancer, metastatic implants

Women with peritoneal endometriosis can be asymptomatic; on the other hand deep pelvic endometriosis is frequently associated with pelvic pain, dysmenorrhea, dyspareunia, urinary tract symptoms, and infertility [12]. Pelvic pain may be chronic rather than cyclic.

The enriched sensory innervation of endometriotic lesions may play a key role in hyperalgesia and pain generation. In deep infiltrating lesions the nerve fibre density is higher than in peritoneal and ovarian ones; in particular, deep infiltrating lesions involving the bowel are the most densely innervated of all lesion types, which correlates with the high incidence of patient-reported pain [13]. According to Koninckx the intensity of the pain is proportional to the depth to which the lesions penetrate [6]; nevertheless, in many cases the extent of endometriotic lesions does not correlate with the severity of symptoms [14].

Clinically, endometriosis should be considered in any woman of reproductive age with pelvic pain or infertility. However, since symptoms are often nonspecific, the disease may be misdiagnosed as other clinical conditions characterized by chronic pelvic pain (irritable bowel syndrome, interstitial cystitis/painful bladder syndrome, recurrent cystitis-overactive bladder), thus leading to inadequate treatment and considerable diagnostic delay. The interval between the onset of the first symptoms and the clinical diagnosis of endometriosis may be of approximately 7–10 years [7].

Clinical manifestations depend on the anatomic locations of the disease.

- *Bladder*: dysuria, gross hematuria during menses, irritative voiding symptoms, urgency, frequent urination, urinary storage symptoms, tenesmus, burning sensation, suprapubic discomfort and pain, urinary incontinence [2, 3, 15].
- *Ureters*: dysmenorrhea, dyspareunia, urinary symptoms, hydronephrosis, flank pain, decline of renal function [2, 3].
- *Round ligaments*: painful, palpable inguinal mass (extra-pelvic portion of the ligaments); nonspecific pelvic pain (intra-pelvic portion) [11].
- *Retrocervical region and uterosacral ligaments*: severe and painful symptoms, dyspareunia [3].
- *Vagina*: dysmenorrhea, dyspareunia, postcoital spotting, prolonged menstruation not responding to medical therapy leading to anaemia [3, 16].
- *Rectosigmoid colon*: cyclic pain during defecation, dyschezia, cyclic hematochezia, bloating, constipation, bowel cramping, catamenial diarrhoea, pencil-like stools, bowel obstruction [2, 3, 12, 17].

When unusual locations outside the pelvis occur, the pain may be site specific.

- *Thoracic-diaphragmatic endometriosis*: chest pain (diffuse or basithoracic) with right-sided predominance, scapular or cervical pain associated with menses, sometimes radiating to the arm, pneumothorax, dyspnea, hemoptysis [18–20].
- *Sciatic nerve*: cyclic sciatica, back pain, gluteal pain radiating to the dorsal thigh and lateral lower leg, positive Lasègue’s sign, sensory loss, reflex alterations, muscle weakness, paresis [2, 21–23].

## Our MR protocol

The MR imaging protocol we use at our institution to study patients with pelvic endometriosis is summarized in Table 2. MR imaging is performed with a closed-configuration superconducting 1.5-T system (Signa HDxT; GE Healthcare, Milwaukee, WI, USA), by using an eight-channel high-resolution surface phased-array torso coil with array spatial sensitivity technique (ASSET) parallel acquisition.

**Table 2:** MRI protocol. Synoptic table summarizes the imaging parameters of MR sequences. Axial T2-weighted SSFSE sequence is used as second localiser to identify the longitudinal axis of the uterus. Sagittal T2-weighted FRFSE sequence is oriented parallel to the longitudinal axis of the uterus identified on the previous axial T2-weighted SSFSE sequence. Oblique coronal and oblique axial T2-weighted FRFSE sequences are oriented respectively parallel or perpendicular to the longitudinal axis of the uterus

MRI protocol	Axial T2 W SSFSE	Sagittal T2 W FRFSE	Oblique coronal/axial T2 W FRFSE	Axial T2*	Axial DWI SE EPI	Sagittal, oblique coronal/axial T1 W 3D GRE LAVA
Repetition time/Echo time (ms)	765/59	4675/100	4675/100	650/11.8	3000/74.1	4.4/2.1
Flip angle	90°	90°	90°	20°	90°	12°
Section thickness (mm)	6	4	4	5	5	3.4
Interslice gap (mm)	0.6	0.4	0.4	0	1	−1.7
Bandwidht (kHz)	31.25	41.67	41.67	8.33	250	62.5
Field of view (cm)	38	32	32	25	46	40
Matrix	320 × 288	320 × 224	320 × 224	256 × 256	160 × 160	320 × 192
N. of averages	0.54	4	4	1	16	0.75
N. of images	30	26	26	30	28	104
Frequency direction	Right to left	Anterior to posterior	Right to left	Anterior to posterior	Anterior to posterior	Superior to inferior
Acquisition time	24 s	3 min 49 s	3 min 49 s	1 min 28 s	3 min 18 s	22 s
b-value (s/mm^2^)	–	–	–	–	0–800	–

Sagittal, oblique coronal and oblique axial T1-weighted 3D gradient-echo liver acquisition with volume acceleration (LAVA) sequences with fat suppression are acquired before and after intravenous administration of paramagnetic contrast agent (0.1 mmol/kg at a flow rate of 2 mL/s, followed by 20 mL of saline solution at the same flow rate); in particular, the sagittal sequence is acquired at 60 and 120 s after contrast administration. About 10 min after contrast administration, T1-weighted 3D gradient-echo LAVA sequences are acquired in the sagittal, coronal and axial planes, to obtain an urographic phase

T2-weighted FRFSE, T2* and DW sequences are acquired with patient breathing freely, T1-weighted 3D gradient-echo LAVA sequences are acquired in breath hold. T2* and DW sequences are not routinely performed and represents optional sequences

*T2 W* T2-weighted, *T1 W* T1-weighted, *SSFSE* single-shot fast spin-echo, *FRFSE* fast relaxation fast spin-echo, *DWI* diffusion-weighted imaging, *SE* spin-echo, *EPI* echoplanar imaging, *GRE* gradient-echo, *LAVA* liver acquisition with volume acceleration

In preparation for imaging, it is recommended that patients fast (4–6 h) before the examination. Bowel preparation includes an enema administered approximately 2–3 h before the examination. The study should not be conducted during the menstrual cycle.

MR imaging is performed with moderate repletion of the patient’s bladder, since an overfilled bladder may cause detrusor contractions and may obliterate the adjacent recesses thus compromising the identification of small parietal nodules [1, 2]. On the other hand, an empty bladder prevents optimal visualization of the ureters.

MR imaging is performed with the patient lying in the supine position (entry position feet first). In patients who show a dilatation of the excretory system, the urographic phase is acquired in the prone position. In claustrophobic patients, prone position may reduce anxiety and improve exam acceptability.

When the clinical evaluation suggests a rectosigmoid endometriosis, rectal opacification is performed before the examination. Retrograde distension of the rectum and the sigmoid colon is obtained inside the gantry with a rectal enema of 750 mL of saline solution introduced through a Nelaton catheter (20 Ch, 6.67 mm × 360 mm). Bowel cleansing is performed through oral administration of a polyethylene glycol solution (1000 mL) the day before the study. In these patients the intravenous administration of an antispasmodic agent, scopolamine-N-butyl bromide (Buscopan® 20 mg; Boehringer Ingelheim, Milano, Italy) just before image acquisition is mandatory to reduce motion artefacts caused by bowel peristalsis. Even if rectal opacification is not strictly necessary to detect endometriotic lesions of the intestinal wall, rectal distension may be useful to evaluate the degree of bowel stenosis.

The state-of-the-art MR imaging protocol for the diagnosis of endometriosis includes T2- and fat suppressed T1-weighted sequences.

*T2-weighted sequences without fat-suppression* are the best sequences for detecting pelvic endometriosis [1], in particular for the evaluation of fibrotic lesions [2].

*Fat-suppressed T1-weighted 3D gradient-echo LAVA sequence.* This pulse sequence improves the sensitivity of MR imaging in the detection of small lesions. It is the most sensitive for the detection of bloody foci and peritoneal endometriosis, since fat suppression narrows the dynamic signal range, thus accentuating the differences in tissue signal. It also increases MRI specificity, since fat-containing lesions such as dermoids are ruled out from the differential diagnosis of endometriomas [14]. LAVA is a fast MR imaging sequence (acquisition time about 22 s), thus, when possible (cooperating patients), acquisition in the three spatial planes allows achieving better anatomical localization of small peritoneal implants, without prolonging too much the examination time.

**Fig. 2:**
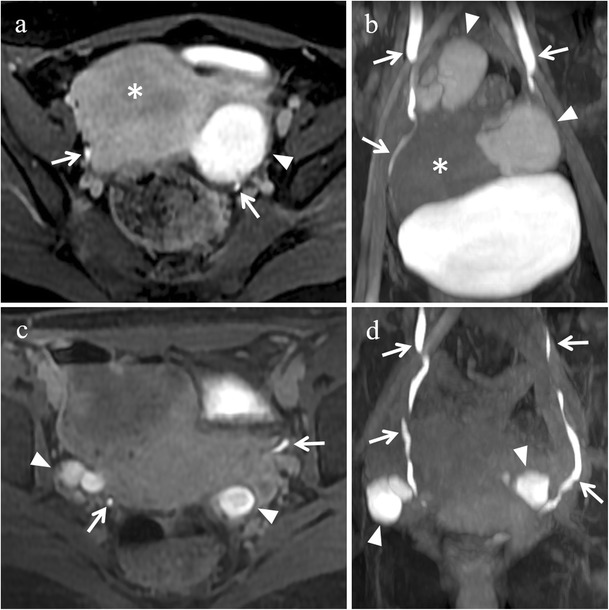
Ureter visualization with intravenous gadolinium and variability of the ureteral course in two different patients with bilateral endometriomas. 27-year-old woman **(a, b)**. **(a)** Axial contrast-enhanced fat-suppressed T1-weighted image (acquired about 10 min after contrast administration) and **(b)** corresponding coronal MIP image. The right ureter (white arrows) courses along the lateral margin of the right endometrial cyst (white arrowhead) and the uterus (white *), the left ureter (white arrows) courses along the posterior margin of the left endometrioma (white arrowheads). 35-year-old woman **(b, c)**. **(c)** Axial contrast-enhanced fat-suppressed T1-weighted image (acquired about 10 min after contrast administration) and **(d)** corresponding coronal MIP image. The right ureter (white arrows) courses medially to the omolateral endometrial cyst (white arrowheads), the left ureter (white arrows) courses laterally to the omolateral endometrioma (white arrowheads). Preoperative knowledge of ureteral course is important in order to prevent iatrogenic injuries to the ureters

*Contrast-enhanced fat-suppressed T1-weighted 3D gradient-echo LAVA sequence* is useful in the following conditions:

- detection of enhancing mural nodules within adnexal masses, when atypical features on US or T2-weighted MR sequences suggest potential malignancy [1];
- in our experience the major benefit of intravenous gadolinium is ureter visualization. The anatomical relationship between the ureters and endometrial cysts or implants is important for the surgeon even when endometriotic lesions do not involve directly the ureters (Fig. 2). Indeed, because of their position from the lateral edge of the cervix, the ureters are particularly vulnerable to detachment or ligation during gynaecological surgery. Injury to the ureters is the most common urologic complication of pelvic surgery, with an incidence that ranges from 1% to 10%, most cases related to gynecologic procedures [24].

Some optional sequences, such as diffusion-weighted and susceptibility-weighted, may be acquired in selected cases according to the authors’ experience.

T2*-weighted sequences depict magnetic susceptibility effects as signal voids [25]. Because of its sensitivity to hemosiderin, this pulse sequence is useful in the detection of old hemorrhagic content of endometriomas and endometriotic implants. Nevertheless, because of the susceptibility artefacts caused by intestinal gas, it is not routinely used in the standard MR protocol and represents an optional sequence.

The potential role of *diffusion-weighted sequences* is described from time to time in the specific subsections of “MR imaging findings”.

## Types of endometriotic lesions: MR imaging appearance with pathologic correlation

The main different types of endometriotic lesions are as follows:

- *endometrial ovarian cysts (endometriomas);*
- *small superficial peritoneal implants;*
- *adhesions;*
- *deep infiltrating endometriosis—solid deep lesions involving round ligaments, parametrium, retrocervical region, USL;*
- *deep infiltrating endometriosis—visceral solid endometriosis involving the bladder and rectal wall.*

In order to understand signal intensity features of endometriotic lesions is mandatory to correlate MR imaging findings with histologic appearance. Pathologic appearance of endometriosis depends on the duration of the disease and depth of penetration of the lesions [14]. The ectopic endometrium responds to hormonal stimulation with cyclic haemorrhage that induce an inflammatory response and fibrous reaction; therefore, at histologic examination endometriotic lesions are characterized by endometrial glands and stroma with various amounts of inflammation and fibrosis. At MR imaging the signal intensity of endometriotic lesions is a function of the quantity and age of the haemorrhage on the one hand and the proportion of endometrial cells and stroma on the other [21].

*Endometrial ovarian cysts (endometriomas)* are discussed in the specific section.

*Small superficial peritoneal implants* (<1 cm in diameter) are the first grossly recognizable lesions on the surface of pelvic organs or pelvic peritoneum. The lesions have a micronodular or microcystic appearance; however, cysts do not enlarge except in the ovary. Only pigmented lesions can be detected at non-contrast-enhanced MR imaging because of the presence of haemorrhage [26]. At MR imaging these small implants manifest as multiple round (cystic or nodular) lesions homogeneously hyperintense on fat-suppressed T1-weighted images, due to old hemorrhagic content, regardless of their signal intensity on T2-weighted images [26].

Involvement of peritoneal reflections over the cul-de-sac and the uterus may also manifest on contrast-enhanced fat-saturated T1-weighted images as diffuse peritoneal enhancement secondary to the inflammatory reaction induced by endometrial implants [26].

Over time a fibrotic reaction may occur, thus leading to adhesions formation between pelvic structures [26].

**Fig. 3:**
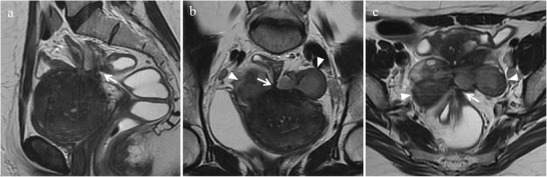
Adhesions in a 40-year-old woman with dysmenorrhea, who underwent two previous surgical interventions for endometriosis. **(a)** Sagittal, **(b)** oblique axial, and **(c)** oblique coronal T2-weighted images show spiculated hypointense strands arranged in confluent angles (white arrows) with loss of the cleavage planes among the anterior surface of the sigma, the posterior uterine serosa and bilateral endometriomas (white arrowheads)

**Fig. 4:**
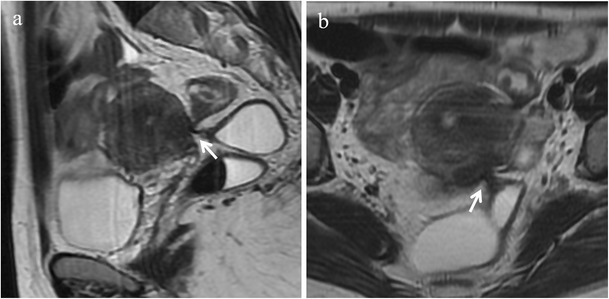
Adhesions in a 43-year-old woman. **(a)** Sagittal, and **(b)** oblique axial T2-weighted images display spiculated hypointense strands (white arrows) between the anterior surface of the sigma and the posterior uterine serosa with angulation of rectosigmoid colon

**Fig. 5:**
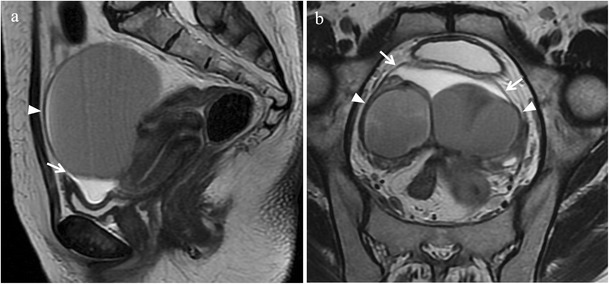
Indirect sign of adhesions in a 36-year-old woman with bilateral endometriomas. **(a)** Sagittal and **(b)** oblique coronal T2-weighted images show free fluid in the pelvis on “anti-declive position” (white arrows), due to the presence of adhesions and bilateral endometriomas (white arrowheads). Both ovaries are joined together involved in adhesions (kissing ovaries)

*Adhesions* represent a common complication of endometriosis. Histologically, they are seen as bands of dense connective tissue mainly composed of type I collagen and poor cellular component represented by fibroblasts and macrophages. At MR imaging, especially on T2-weighted images, retractile adhesions may be observed as spiculated hypointense peritoneal strands arranged in confluent angles (Figs. 3 and 4). Tethering of pelvic structures and loss of the corresponding cleavage planes, without appreciable nodular lesions, may be another imaging finding suggestive of adhesions [27]. Extensive adhesions may distort pelvic anatomy, compartmentalize the pelvis and obliterate Douglas pouch. Posterior displacement of uterus and ovaries, angulation of rectosigmoid colon and bowel loops, elevation of the posterior vaginal fornix, loculated fluid collections, and a hydrosalpinx may be indirect signs of adhesions [14]. Free fluid in the pelvis on “anti-declive position” rather than in the cul-the-sac may be another indirect sign of adhesions (Fig. 5).

In *deep infiltrating endometriosis* endometrial glands and stroma infiltrate the peritoneum and then the adjacent tissues and induce smooth muscle proliferation and fibrous reaction, thus causing the formation of *solid nodules*.

In *visceral solid endometriosis*, implants adhere to the serosal surface of the bladder or intestinal wall and may invade the underlying muscular layers, inducing smooth muscle proliferation and fibrosis, and eventually the submucosa.

At MR imaging both fibrous tissue and smooth muscle show intermediate signal intensity on T1-weighted images and low signal intensity on T2-weighted images. Therefore, on T2-weighted images, solid endometriotic lesions appear as hypointense nodular structures with irregular or stellate margins due to fibrous tissue and smooth muscle proliferation. In certain cases, deep endometriotic lesions may also appear as irregular and hypointense soft-tissue nodular thickening on T2-weighted sequences, as it occurs when the disease involves the USL or the vaginal or rectal wall. Within solid endometriotic masses, hyperintense foci on T2-weighted images may be seen, representing dilated ectopic endometrial glands [2].

**Fig. 6:**
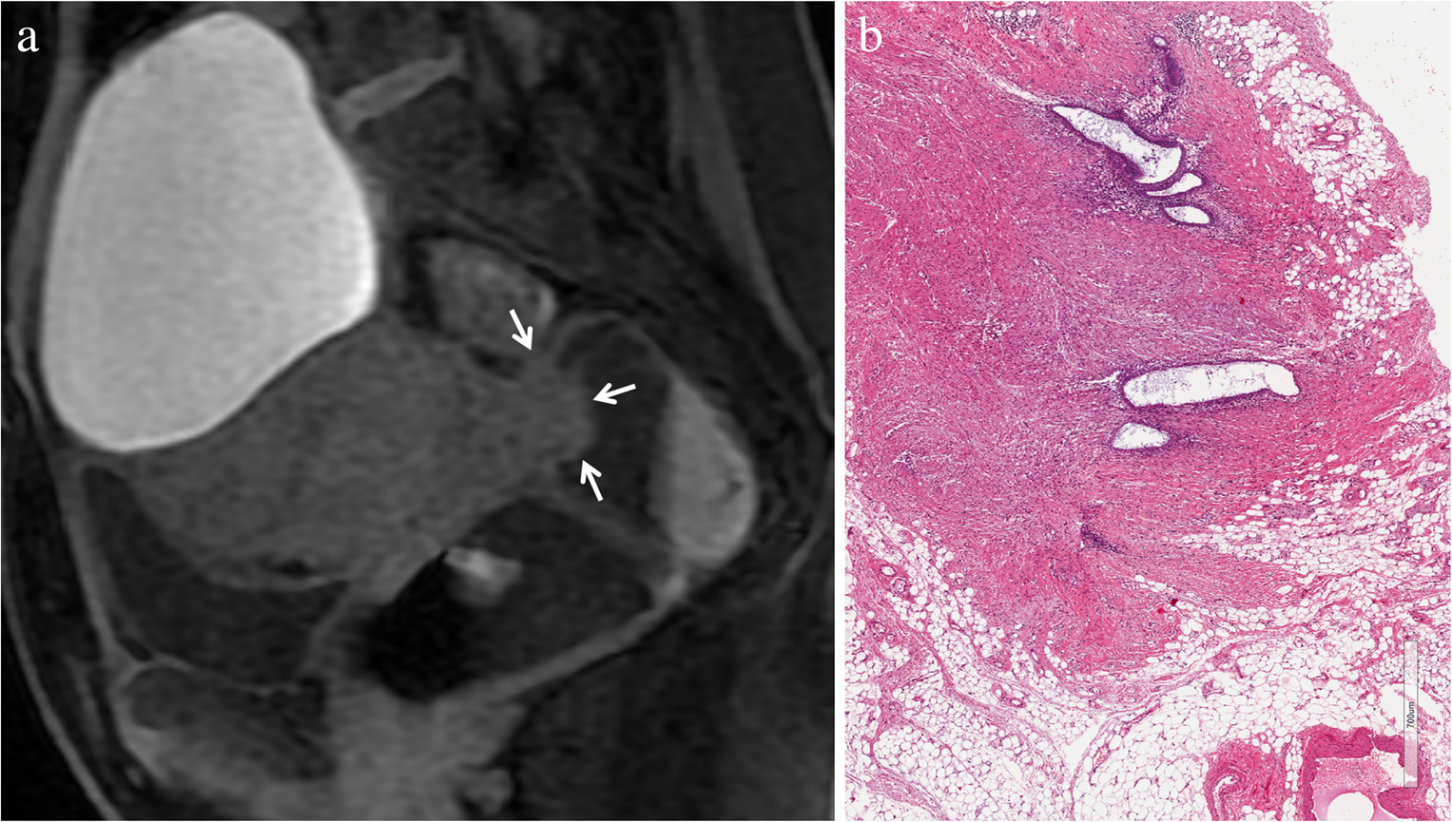
Deep infiltrating endometriosis in a 34-year-old woman. **(a)** Sagittal fat-suppressed T1-weighted image shows an endometriotic nodule (white arrows) infiltrating the muscular layer of the anterior rectal wall. The lesion displays homogeneous intermediate signal intensity due to fibrous tissue and smooth muscle. **(b)** Photomicrograph (H&E 250X). Focus of endometriosis without haemorrhage

**Fig. 7:**
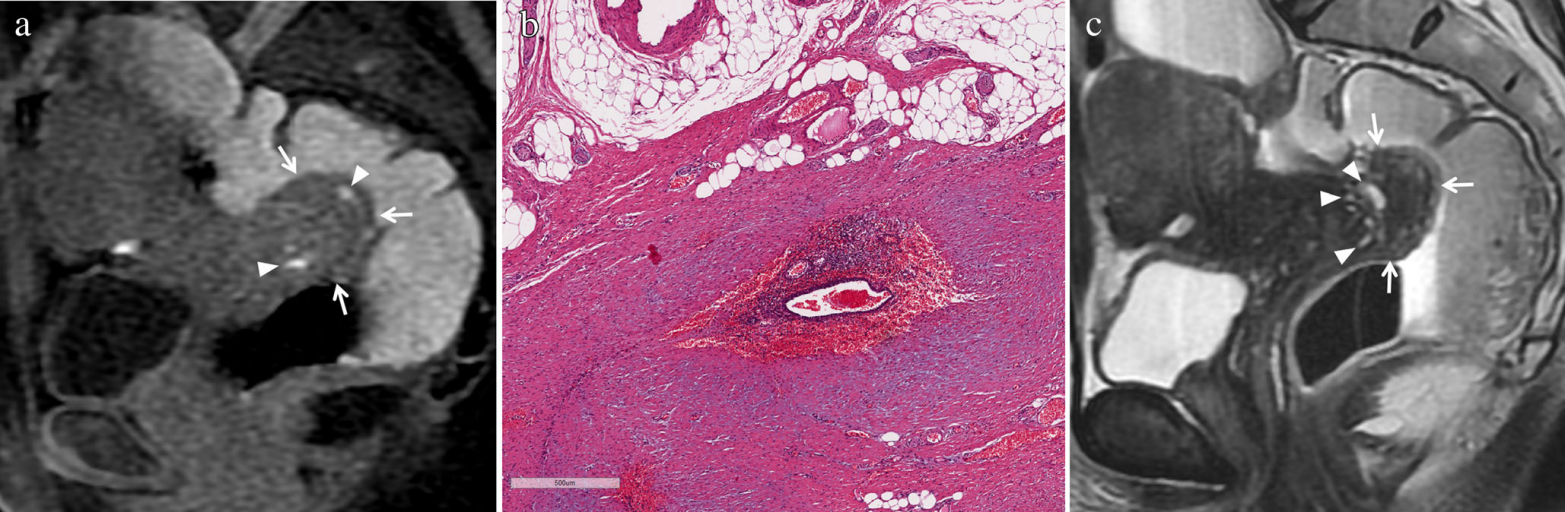
Deep infiltrating endometriosis in a 48-year-old woman. **(a)** Sagittal fat-suppressed T1-weighted image displays an endometriotic nodule (white arrows) infiltrating the muscular layer of the anterior rectal wall. Within the lesion hyperintense foci are detectable (white arrowheads), representing hemorrhagic content. **(b)** Photomicrograph (H&E 200×). Focus of endometriosis with marked haemorrhage. On **(c)** sagittal T2-weighted image the solid endometriotic lesion appears mainly hypointense (white arrows), with small hyperintense cystic foci inside representing dilated ectopic endometrial glands (white arrowheads)

Usually, endometrial glands without haemorrhage are not detectable on fat-suppressed T1-weighted images; so deep lesions may show homogeneous intermediate signal intensity on T1-weighted images (Fig. 6). When red cell extravasation outside the glandular ducts into the surrounding stroma occurs, these small haemorrhages become visible as small hyperintense spots on fat saturated T1-weighted images (Fig. 7).

However, unlike endometriomas and superficial peritoneal implants, deep infiltrating visceral solid endometriosis (especially of the rectal wall) is less likely to contain hyperintense foci on fat saturated T1-weighted images, probably because surrounding fibrous reaction and smooth muscle proliferation minimize cyclical bleeding within ectopic endometrial glandular ducts [28].

In certain locations (i.e. round ligaments) the absence of the hyperintense hemorrhagic foci on T1-weighted images may be due to the effect of hormonal treatment [11].

After the intravenous administration of gadolinium, lesion enhancement may occur due to inflammatory reaction, glandular and fibrous tissue.

## MR imaging findings

Because of the multifocal nature of the disease, often foci of endometriosis are simultaneously observed at different sites. Owing to the possibility to perform a complete assessment of all pelvic compartments at one time [12], MRI represents the best imaging technique for preoperative staging of endometriosis.

### Urinary tract

Endometriotic lesions may affect the urinary tract in up to 20% of cases; these implants are associated with lesions in other pelvic locations in up to 50–75% of cases [2, 14]. The bladder is the most frequently involved organ (85%), followed by ureter (9%), kidney (4%), and urethra (2%) [29]. Different locations of urinary tract endometriosis may coexist, especially the bladder and ureters. Bladder endometriosis is often multifocal, the trigone and the dome being the most frequently affected sites [15]. The distal ureter, 3–4 cm above the vesico-ureteral junction, is the most common ureteral segment involved [14, 30].

According to the degree of wall infiltration, bladder and ureteral involvement may be classified as extrinsic or intrinsic. In extrinsic involvement, the most common form, implants are confined to the serosal surface (or the ureteral adventitia) or the surrounding connective tissue; it can be treated by bladder or ureteral shaving. In intrinsic involvement lesions infiltrate the muscular layer manifesting as mural masses; rarely implants infiltrate and ulcerate the mucosal layer. Preoperative differentiation between intrinsic and extrinsic ureteral endometriosis is not always easy; furthermore, the two types may coexist.

Regards to its origin, bladder endometriosis may be classified as primary when it occurs spontaneously, or as secondary when it is related to iatrogenic lesions due to pelvic surgery (caesarean delivery, hysterectomy) [31].

**Fig. 8:**
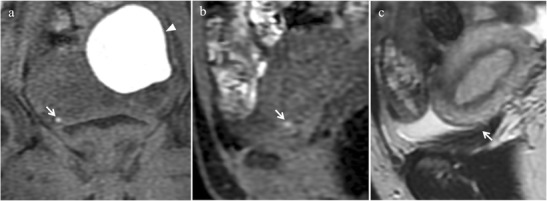
Bladder endometriosis with extrinsic involvement in a 21-year-old woman with left endometrioma and other endometriotic implants involving the left fallopian tube, the uterine serosa and the right uterosacral ligament. **(a)** Coronal and **(b)** sagittal fat-suppressed T1-weighted images demonstrate one spot of high signal intensity on the serosal surface of the bladder representing a small peritoneal implant with hemorrhagic content (white arrows). Note the endometrioma in the left ovary (white arrowhead in a). On **(c)** sagittal T2-weighted image the implant (white arrow) is hardly detectable because it is partly masked by the hypointense signal of the bladder wall

**Fig. 9:**
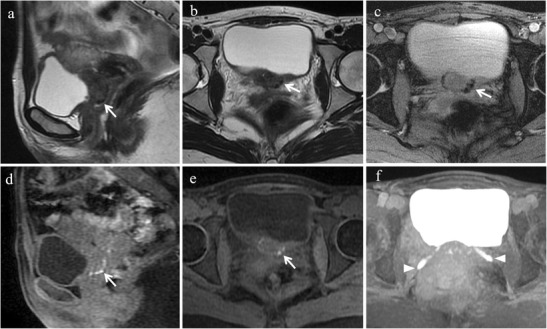
Bladder endometriosis with intrinsic involvement in a 35-year-old woman with dysuria, hematuria and urinary incontinence during menses. **(a)** Sagittal and **(b)** oblique axial T2-weighted images show a mural mass with low signal intensity infiltrating the posterior bladder wall (white arrows). On **(c)** axial T2* image punctate signal voids due to hemosiderin deposition can be seen along the borders of the lesion (white arrow). On **(d)** sagittal and **(e)** oblique axial fat-suppressed T1-weighted images the implant displays intermediate signal intensity with spots of high signal intensity, representing hemorrhagic content (white arrows). **(f)** Oblique axial MIP image (acquired about 10 min after contrast administration) demonstrates the relationship between the lesion and the ureters (white arrowheads)

At MR imaging bladder endometriosis may manifest as localized or diffuse wall thickening and signal intensity abnormalities [12]. The appearance is of low signal intensity on T2-weighted and intermediate signal intensity on T1-weighted images, with or without spots of high signal intensity on T1-weighted images, representing hemorrhagic content [32] (Figs. 8 and 9). Implants minimally enhance after injection of a gadolinium-based contrast material. The maximum lesion diameter varies between 1 and 5 cm. MRI reaches sensitivity up to 88%, specificity up to 99% and diagnostic accuracy of about 98% for the diagnosis of bladder endometriosis [15].

Clinical management of bladder endometriosis may be conservative, using hormonal therapies, or surgical. For planning a correct surgical treatment, it is important: to ascertain the precise location of bladder nodule (distance between the ureteral meatus and the caudal border of the endometriotic lesion) and to define the ureteral status [15].

The differential diagnosis of bladder endometriosis includes urachal remnant, epithelial tumours (bladder carcinoma) and mesenchymal tumours (angiomas, leiomyoma) [3, 15]. Malignant transformation of bladder endometriosis is extremely rare [31].

Ureteral endometriosis may be defined as any situation where endometriosis or surrounding associated fibrosis causes compression or distortion of the normal ureteral anatomy, even when hydroureteronephrosis is not yet present [33, 34]. All patients with urinary tract endometriosis should be assessed for renal function by blood creatinine measurements, since when endometriosis involves the ureters a silent loss of renal function may occur [15, 29]. Ureteral endometriosis is most often unilateral, with a left predisposition; bilateral involvement is present in approximately 10–20% of cases [30]. Ureteral involvement is often associated with an ipsilateral endometrioma or with a recto-vaginal nodule larger than 3 cm [29, 30]. Even if ureteral endometriosis is now increasingly recognized because of the improvement in diagnostic tools [35], preoperative diagnosis is challenging mostly when it is not complicated by obstruction and proximal dilatation [36].

**Fig. 10:**
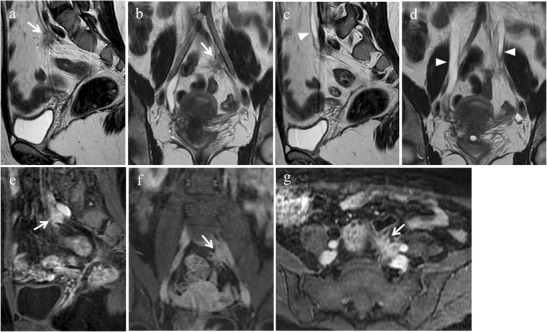
Ureteral endometriosis in a 35-year-old woman with multiple DIE lesions. **(a)** Sagittal and **(b)** coronal T2-weighted images show a retroperitoneal solid nodule with spiculated margins (white arrows), with low signal intensity, adjacent to left iliac vessels. **(c)** Sagittal and **(d)** coronal T2-weighted images demonstrate dilatation of the ureter upstream and of the contralateral ureter (white arrowheads). On **(e)** sagittal, **(f)** coronal and **(g)** axial contrast-enhanced fat-suppressed T1-weighted images the lesion displays enhancement (white arrows)

MR is the best imaging technique for ureteral evaluation. On T2-weighted MR images ureteral implants appear as solid nodules with spiculated margins, showing low signal intensity, that envelop the ureter, causing dilatation of the ureter upstream [3] (Fig. 10).

In a retrospective series including 77 patients affected by ureteral endometriosis Seracchioli et al. [30] described two different histological pattern of ureteral involvement: endometriotic ureteral endometriosis characterized by endometrial glands and/or stroma cells within the wall of the ureter or within periureteral tissue, and fibrotic ureteral endometriosis where only fibrosis tissue was observed. They found that the endometriotic pattern (77% of patients) was significantly associated with hydroureteronephrosis at pre-operative uro-CT scan, whereas the fibrotic pattern (23% of patients) was significantly associated with concomitant endometriosis in the recto-vaginal septum. According to author’s point of view this new classification of ureteral endometriosis based on tissue composition and histological pattern could be useful in both diagnostic and therapeutic fields. Indeed, fibrotic tissue typically does not respond to hormonal therapy [35].

When deep pelvic endometriosis involves the uterosacral ligaments and rectovaginal septum, ureteral involvement may occur even if not suggested preoperatively. Hence these patients should undergo retroperitoneal laparoscopic inspection and isolation of both ureters in order to avoid complications [36].

The differential diagnosis of ureteral endometriosis includes ureteral invasion by cervical cancer.

### Ovaries

The ovaries are the most common site of endometriosis (20–40% of cases) [3].

Ovarian endometriosis may show the following patterns [37]:

- superficial implants associated with fibrous adhesions;
- micro intra-ovarian endometriomas;
- deep implants with repeated cyclic haemorrhage resulting in endometriotic cysts (endometriomas).

Peritoneal implants confined to the ovarian surface are often underdiagnosed at imaging due to their small size (< 5 mm) [37].

Micro intra-ovarian endometriomas are small size (< 1 cm) implants within the ovaries. They show hyperintense signal on T1-weighted images and variable signal intensity on T2-weighted images. Often multiple and bilateral, these endometriotic foci may be overlooked at laparoscopy [37].

Endometriomas are pseudocysts with hemorrhagic content, formed by the invagination of endometriosis within the ovarian cortex [9]; they are frequently multilocular and bilateral (50% of cases).

**Fig. 11:**
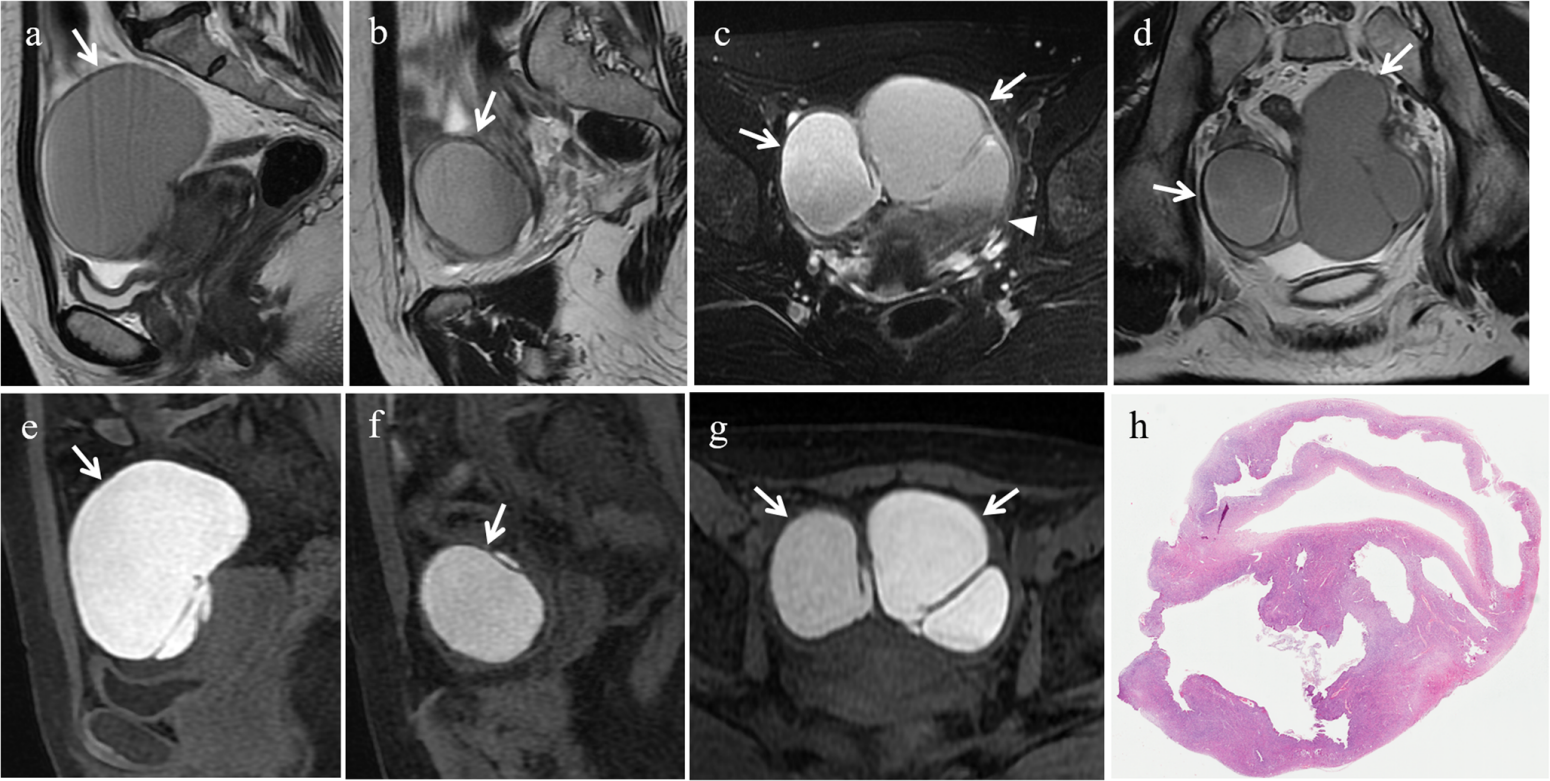
Ovarian endometriotic cysts in a 36-year-old woman. The same patient as in Fig. 5. **(a, b)** Sagittal, **(c)** oblique axial and **(d)** oblique coronal T2-weighted images show bilateral endometriomas with intermediate to low signal intensity (white arrows). The ovaries are joined together behind the uterus (kissing ovaries). Note the low signal intensity in the declivous portion of the left cyst (“shading” sign, white arrowhead in c). On **(e, f)** sagittal and **(g)** oblique axial fat-suppressed T1-weighted images the cysts demonstrate high signal intensity (white arrows). **(h)** Photomicrograph (H&E 20X). Ovarian endometriotic cyst

Even though in the differential diagnosis of ovarian endometriosis, transvaginal US is highly sensitive and specific, MR imaging is considered by some authors as the best imaging modality for diagnosing endometriomas [3]. At MR imaging the pathognomonic feature of endometriomas is the “shading” sign, which can be seen on T2-weighted images. It reflects the chronic nature of endometriomas and is the result of cyclic bleeding occurring over time. Old blood products contain high iron and protein concentrations which determine a decrease in T2-relaxation time. Therefore, on T2-weighted images endometriomas will show a gradual loss of signal within the lesion with low signal intensity till complete signal void in the declivous portion (“shading”). Endometriotic cysts show high signal intensity on T1-weighted images. Fluid-fluid levels may also be observed within the lesion [3, 14, 38, 39] (Fig. 11).

The most specific pathologic feature of endometrioma is the thick fibrous capsule containing a cluster of hemosiderin-laden macrophages due to repeated haemorrhage. Takeuchi et al. [25] evaluated hemosiderin deposition within the walls of endometriomas on susceptibility-weighted MR images at both 3.0 T and 1.5 T. They found punctate or curved linear signal voids due to hemosiderin deposition along the cyst wall in 92.9% of the endometriomas of their series, thus concluding that this imaging sign may be diagnostic of endometrioma and that susceptibility-weighted imaging can contribute to its diagnosis.

In certain cases, the ovaries may be joined together behind the uterus in the pouch of Douglas due to adhesion formation between the adjacent peritoneal surfaces, a sign described at US as “kissing ovaries” and suggestive of severe pelvic endometriosis [40].

In a recently published meta-analysis the sensitivity and specificity of MRI for the diagnosis of endometrial cysts were 95% and 91% respectively [41].

The differential diagnosis of endometriomas includes lesions with high signal intensity on T1-weighted images: dermoids, mucinous cystic neoplasms, and hemorrhagic masses.

Fat saturated T1-weighted sequences are helpful to rule out a fat-containing lesion (such as dermoids) and to confirm the presence of blood [39].

Mucinous lesions may show hyperintensity on T1-weighted images, but signal intensity is lower than that of blood.

Hence, the most challenging differential diagnosis is with other hemorrhagic masses. To differentiate endometriomas from functional hemorrhagic cysts is important in order to prevent unnecessary surgical interventions. Functional hemorrhagic cysts (i.e. hemorrhagic follicular cysts and hemorrhagic corpus luteum cysts) are usually unilocular and unilateral, do not display shading on T2-weighted images, and mostly disappear on follow-up examinations (generally in 4–6 weeks) [14].

The role of DWI sequences in differentiating endometriomas from functional hemorrhagic ovarian cysts is still debated. Balaban et al. [42] found significantly lower ADC values in endometriomas compared with functional hemorrhagic ovarian cysts in all b values. On the other hand, in a retrospective study by Lee et al. [43] the mean ADC values of endometriomas (1.06 ± 0.38 × 10^−3^ mm^2^/s) was significantly higher than that of functional hemorrhagic cysts (0.73 ± 0.29 × 10^−3^ mm^2^/s).

Endometrioid and clear cell tumours may be associated with endometriosis. Large lesions with wall nodularity, thick septations and enhancing solid components may be suggestive of malignancy.

Surgical excision of an ovarian endometrioma is effective in managing pain. Nevertheless, operative treatment of endometriomas is controversial in women desiring future fertility since it may lead to reduced ovarian reserve in the short term and premature ovarian insufficiency; bilateral and recurrent endometrioma excision may further reduce ovarian reserve [44].

### Uterine serosa, round ligaments, broad ligaments, fallopian tube

The vesicouterine pouch or anterior cul-de-sac is a common site of endometriotic involvement [2]. Either peritoneal or deep endometriotic implants involving the serosal surface of the uterus often determine adhesions between the peritoneal folds of the bladder dome and the uterus with anteflexion of the uterus and obliteration of the anterior cul-de-sac.

**Fig. 12:**
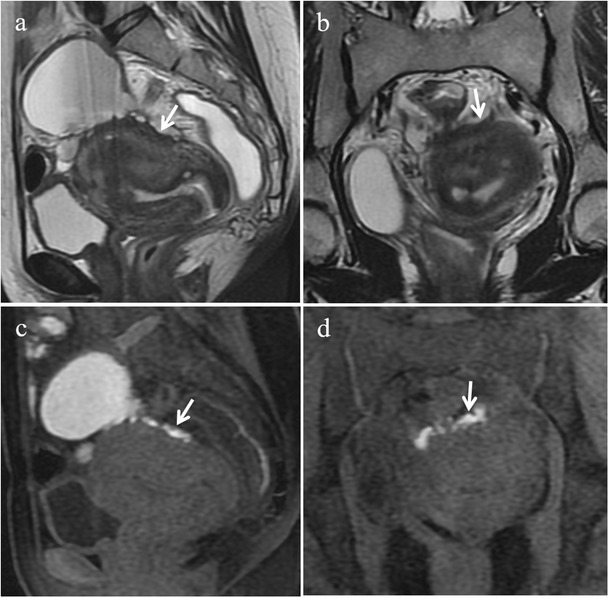
Endometriosis of the uterine serosa in a 46-year-old woman, who underwent a previous surgical intervention for endometrioma of the right ovary. **(a)** Sagittal and **(b)** oblique axial T2-weighted images show endometriotic implants involving the uterine serosa. The lesions demonstrate indistinct margins and low signal intensity (white arrows). On **(c) s**agittal and **(d)** coronal fat-suppressed T1-weighted images the lesions display high signal intensity (white arrows)

At MR imaging deep endometriotic implants involving the anterior uterine serosa demonstrate infiltrative pattern with indistinct margins and show low signal intensity on T2-weighted images, with small cystic areas (Fig. 12) [3].

At MRI the round ligaments can be identified as thin structures with hypointense fibrous signal on T1- and T2-weighted images, extending from the uterine horns to the pelvic sidewall, passing anteriorly to the external iliac vessels. They have an intra- and an extra-pelvic portion, the latter being the distal part of the ligament in the canal of Nuck [11].

**Fig. 13:**
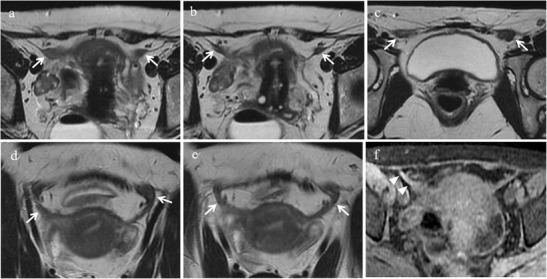
Endometriosis of the round ligaments in a 45-year-old woman with dysmenorrhea, urinary symptoms and chronic pelvic pain, who underwent a previous surgical intervention for endometrioma of the left ovary. **(a-c)** Oblique coronal and **(d, e)** oblique axial T2-weighted images. Both round ligaments (white arrows) appear thickened, nodular and shortened. **(f)** Oblique coronal fat-suppressed T1-weighted image reveals small intralesional high signal-intensity foci within the right ligament (white arrowheads) that represent hemorrhagic component

When involved by endometriosis, round ligaments appear thickened (more than 1 cm), nodular, shortened and irregular (Fig. 13). Usually endometriotic implants are a mixture of fibrous tissue and haemorrhage. Fibrous tissue shows hypointense signal on T1- and T2-weighted images; small hemorrhagic foci displays hyperintense signal on fat-suppressed T1-weighted images [11]. The presence of free fluid around the intra-pelvic portion of the round ligaments may represent an indirect sign of endometriosis [11].

Endometriosis of the broad ligaments usually manifests as thickening and nodularity of these peritoneal folds extending between the uterus and the lateral walls of the pelvis [2].

Asymmetry of the morphology or signal intensity at MR imaging of the parametrium may represent a sign of endometriotic involvement [26].

Endometriosis is the most common cause of hematosalpinx and peritubal adhesions in women of reproductive age. Endometriotic involvement of the fallopian tubes is strongly associated with infertility. Serosal or subserosal implants involves the peritoneal surface of the fallopian tubes, where repeated haemorrhages lead to fibrosis and retraction of the tube with hydrosalpinx. Intraluminal implants determine cyclic haemorrhage thus causing hematosalpinx.

**Fig. 14:**
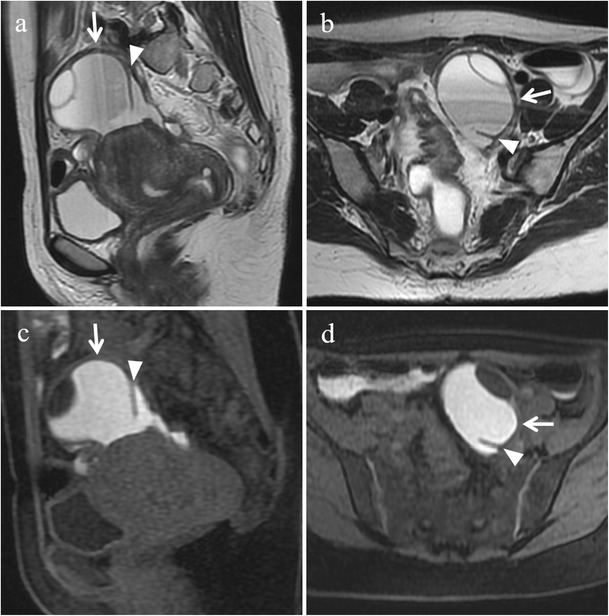
Hematosalpinx in a 46-year-old woman with endometriosis. The same patient as in Fig. 12. **(a)** Sagittal and **(b)** axial T2-weighted images show a tortuous, tubular structure with internal fluid-fluid level in the left adnexa (white arrows). On **(c)** sagittal and **(d)** axial fat-suppressed T1-weighted images endoluminal content displays high signal intensity (white arrows), a finding consistent with hematosalpinx. Note the incomplete mucosal and submucosal plicae along the tubal wall (white arrowheads)

At MR imaging hematosalpinx appears as a tortuous enlarged tubular adnexal structure filled with hemorrhagic fluid. Endoluminal content shows high signal intensity on fat-suppressed T1-weighted images and intermediate signal intensity, with or without internal fluid-fluid level, on T2-weighted images [45] (Fig. 14). According to Siegelman the presence of T1-weighted hyperintensity within a dilated fallopian tube is suggestive of endometriosis [28].

Nevertheless, more rarely hematosalpinx can be associated with other pathologic conditions such as tubal torsion, tubal ectopic pregnancy or malignancy.

### Retrocervical region, uterosacral ligaments

The retrocervical area is a virtual extraperitoneal space behind the cervix, located above the rectovaginal septum [2]. It is a common site of deep pelvic endometriosis.

**Fig. 15:**
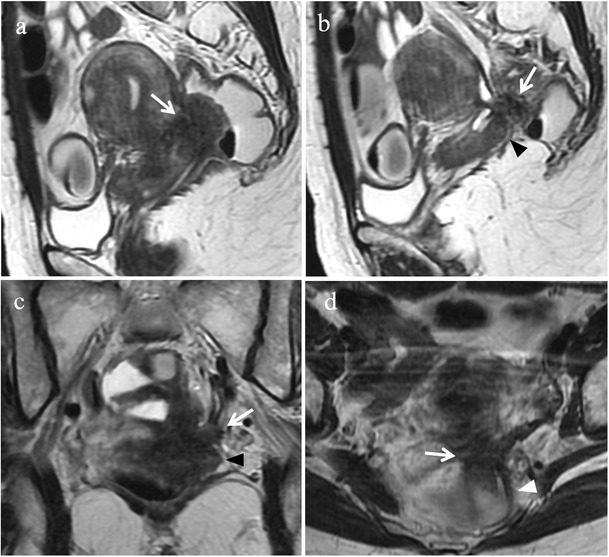
Endometriosis of the retrocervical region in a 39-year-old woman with dysmenorrhea, dyspareunia, tenesmus, catamenial dyschezia and dysuria. **(a, b)** Sagittal, **(c)** oblique coronal and **(d)** oblique axial T2-weighted images show a retrocervical hypointense endometriotic implant (white arrows) infiltrating the posterior uterine serosa and the anterior rectal wall, extending to the left vaginal fornix (black arrowheads in b and c) and to the left uterosacral ligament (white arrowhead in d)

Retrocervical implants are often associated with USL involvement and may extend inferiorly to the posterior vaginal fornix or posteriorly to the anterior rectal wall (Fig. 15). Usually such lesions determine obliteration of the pouch of Douglas and uterine retroflexion. In more severe cases, endometriosis of this anatomical region may determine multiple adhesions and distortion of pelvic anatomy resulting in a frozen pelvis [3].

**Fig. 16:**
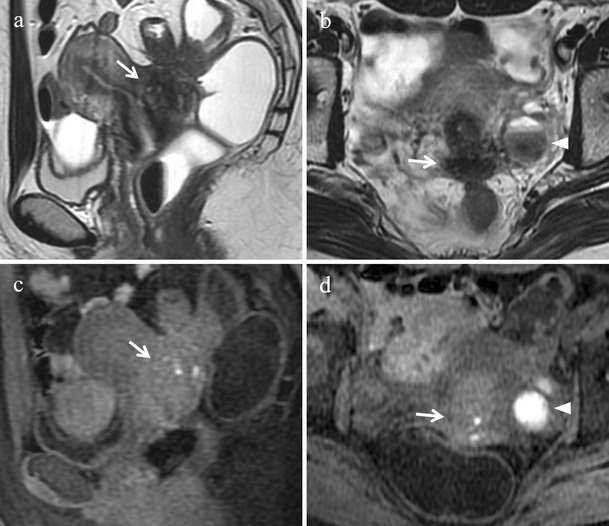
Endometriosis of the retrocervical region in a 41-year-old woman with dysmenorrhea and dyspareunia, who underwent previous laparoscopic adhesiolysis. **(a)** Sagittal and **(b)** oblique axial T2-weighted images show a hypointense ill-defined infiltrative tissue (white arrows) involving the posterior portion of the cervix, the retrocervical region and the anterior rectal wall. Small cystic cavities are seen within the lesion. On **(c)** sagittal and **(d)** axial fat-suppressed T1-weighted images the lesion exhibits intermediate signal intensity (white arrows) with small hyperintense foci reflecting haemorrhage. Note the small left hemorrhagic cyst (white arrowheads in b and d) showing a fluid-fluid level on T2-weighted image; it disappeared in the follow-up US examinations and was likely a hemorrhagic follicular cyst

Deep endometriotic lesions of the retrocervical area frequently appear as ill-defined infiltrative tissue, hypointense on T2-weighted images, extending from the posterior uterine serosa to the retrocervical region [2, 3]. Nevertheless, some lesions may contain abundant glandular component and little fibrotic reaction, thus showing high signal intensity on T1-weighted images and variable signal intensity on T2-weighted images [2, 12]. Small cystic areas, hyperintense on T2-weighted images may also be seen (Fig. 16) [3]. The solid glandular component enhances after intravenous administration of contrast material [2, 12].

In a recently published meta-analysis the sensitivity and specificity of MRI for the diagnosis of endometriosis of the pouch of Douglas were 89% and 94%, respectively [46].

The differential diagnosis of retrocervical lesions includes peritoneal metastases from intraperitoneal malignancies (i.e. gastrointestinal and ovarian neoplasms). Peritoneal metastases usually show intermediate to high signal intensity on T2-weighted images and, as the primary cancer site, high signal intensity on DWI [47]; moreover, ascites and a tumour mass into the abdominal cavity may be identified. On the other hand, solid endometriosis shows low signal intensity on T2-weighted images.

USL are the most frequent location of deep endometriosis. Bilateral USL involvement is often associated with other posterior deep endometriotic locations, mostly the rectosigmoid colon [48].

**Fig. 17:**
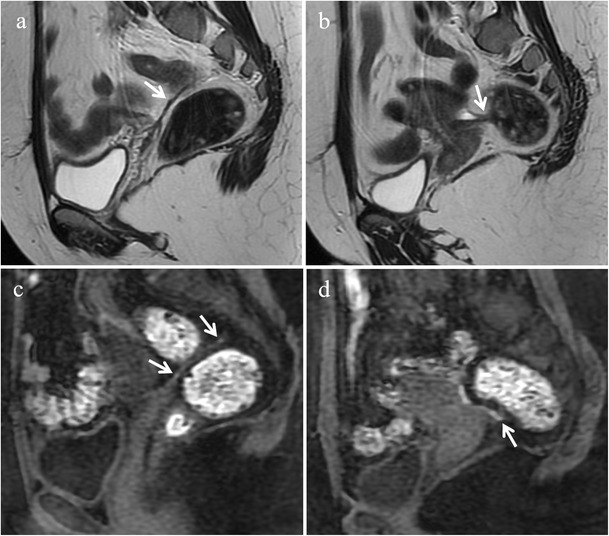
Endometriosis of the uterosacral ligaments in a 35-year-old woman with multiple DIE lesions. The same patient as in Fig. 10. **(a, b)** Sagittal T2-weighted images show hypointense thickening of both uterosacral ligaments (white arrows). **(c, d)** Sagittal fat-suppressed T1-weighted images demonstrate hyperintense spots within the ligaments (white arrows), representing hemorrhagic foci

**Fig. 18:**
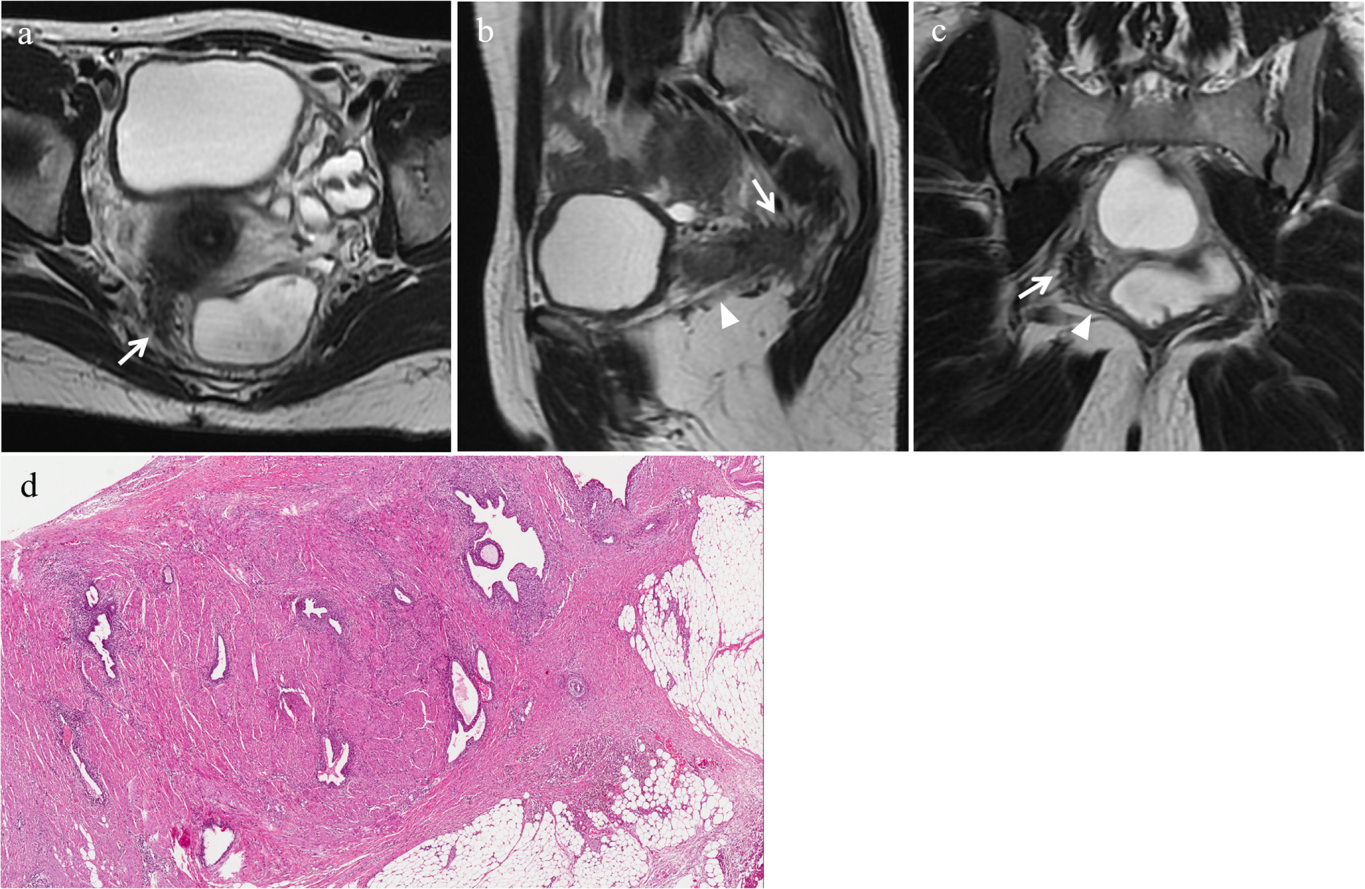
Endometriosis of the right uterosacral ligament in a 28-year-old woman with dyspareunia. **(a)** Oblique axial, **(b)** sagittal and **(c)** oblique coronal T2-weighted images show a low-signal-intensity endometriotic lesion with spiculated margins involving the right uterosacral ligament (white arrows). The lesion extends to the iliococcygeus muscle (white arrowheads). **(d)** Photomicrograph (H&E 250X). Endometriosis in the uterosacral ligament

At MR imaging normal USL are not visible [48] or are depicted as thin, regular, semicircular hypointense cords that originate from the lateral aspect of the uterine cervix and the vaginal vault and course dorsocranially toward the sacrum [2]. USL endometriosis is depicted as nodularity within the ligament or as unilateral or bilateral hypointense thickening of the ligament, with regular or irregular margins [2, 48]. Hyperintense spot on fat-suppressed T1-weighted images, representing punctate foci of haemorrhage, may also be observed [12] (Figs. 17 and 18). The proximal medial portion of the USL is most commonly affected by endometriosis [2]. According to Bazot et al. [48] thin-section oblique axial T2-weighted sequences (3 mm thick, perpendicular to the long axis of the cervix) can improve the capability of conventional MRI to assess USL endometriosis. Left uterosacral endometriosis may be more difficult to diagnose than the right one, because of the frequent location of the rectosigmoid colon in the left part of the pelvic cavity [48].

In a recently published meta-analysis the sensitivity and specificity of MRI for the diagnosis of endometriosis of USL were 85% and 80%, respectively [46].

In patients undergoing surgery, to establish whether USL endometriosis is unilateral or bilateral is particularly relevant. Indeed, the risk of urinary dysfunction and dysuria is significantly higher in patients after bilateral than unilateral USL resection [49]. Other potential complications of the resection of USL are bleeding, ureteral lesions, and pelvic support disorders [50].

### Rectovaginal space

The rectovaginal space is the anatomical region located between the posterior vaginal wall and the anterior rectal wall. It extends from the deepest part of the pouch of Douglas to the top of the perineal body. The inferior two thirds of this space constitute the rectovaginal septum, a thin membranous partition usually filled with fat [2, 3].

Usually rectovaginal implants represent extensions from retrocervical or posterior vaginal lesions, but may also involve the rectovaginal septum alone, without any link to the cervix. These latter implants usually manifest as small nodular lesions palpable at vaginal examination. At MR imaging they show low signal intensity on T2-weighted images [2, 3].

In a recently published meta-analysis the sensitivity and specificity of MRI for the diagnosis of rectovaginal septum endometriosis were 82% and 77%, respectively [46].

### Vagina

Vaginal endometriosis is usually associated with implants in other pelvic locations, mostly retrocervical and rectal lesions; seldom isolated involvement of the vagina may occur. The upper one-third of the vagina and the posterior fornix are the most commonly affected sites.

**Fig. 19:**
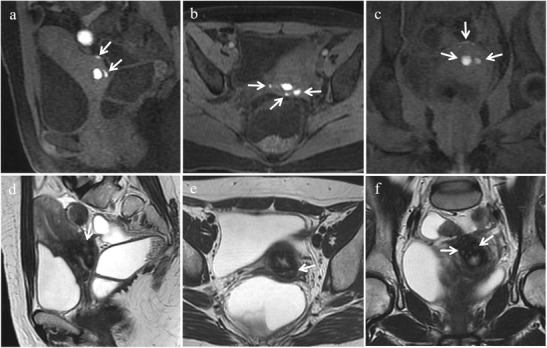
Endometriosis of the vagina and cervix in a 46-year-old woman. **(a)** Sagittal, **(b)** oblique axial, **(c)** oblique coronal fat-suppressed T1-weighted images, and **(d)** sagittal, **(e)** oblique axial and **(f)** oblique coronal T2-weighted images show small endometriotic implants involving the posterior vaginal fornix and the cervix, with multiloculated appearance (white arrows) characterized by cystic areas with hyperintense content, better depicted on fat-suppressed T1-weighted images due to subacute blood products

Generally, the vaginal wall implants show a thickened or nodular appearance [2, 3], but may also have a polypoid structure. Distention of the vaginal lumen with gel may facilitate the identification of the lesion [3]. At MR imaging vaginal endometriotic implants show low signal intensity on T2-weighted images. They often have a multiloculated internal appearance because of the presence of cystic areas. These locules can show hyperintense content on T1-weighted images due to subacute blood products [16] (Fig. 19). Polypoid variant may have a T2 hypointense rim corresponding to surrounding fibrous tissue associated with endometriosis [16]. Rectovaginal fistulation represents a complication of vaginal endometriosis.

In a recently published meta-analysis the sensitivity and specificity of MRI for the diagnosis of vaginal and posterior vaginal fornix endometriosis were 82% and 82%, respectively [46].

Differential diagnosis includes epithelial neoplasms arising from the uterine cervix or vaginal wall. DWI may be useful in this setting demonstrating no restricted diffusion within the endometriotic mass, thus avoiding invasive surgery [16]. On the other hand, vaginal carcinoma displays restricted diffusion on DWI.

### Rectosigmoid colon

Among the bowel segments the rectosigmoid is the most commonly involved by endometriosis (65.7%) [17], followed by vermiform appendix, terminal ileum, cecum and descending colon, in order of frequency [51]. Rectosigmoid endometriosis is often associated with other pelvic locations and with a second intestinal lesion in 55% of cases [3].

**Fig. 20:**
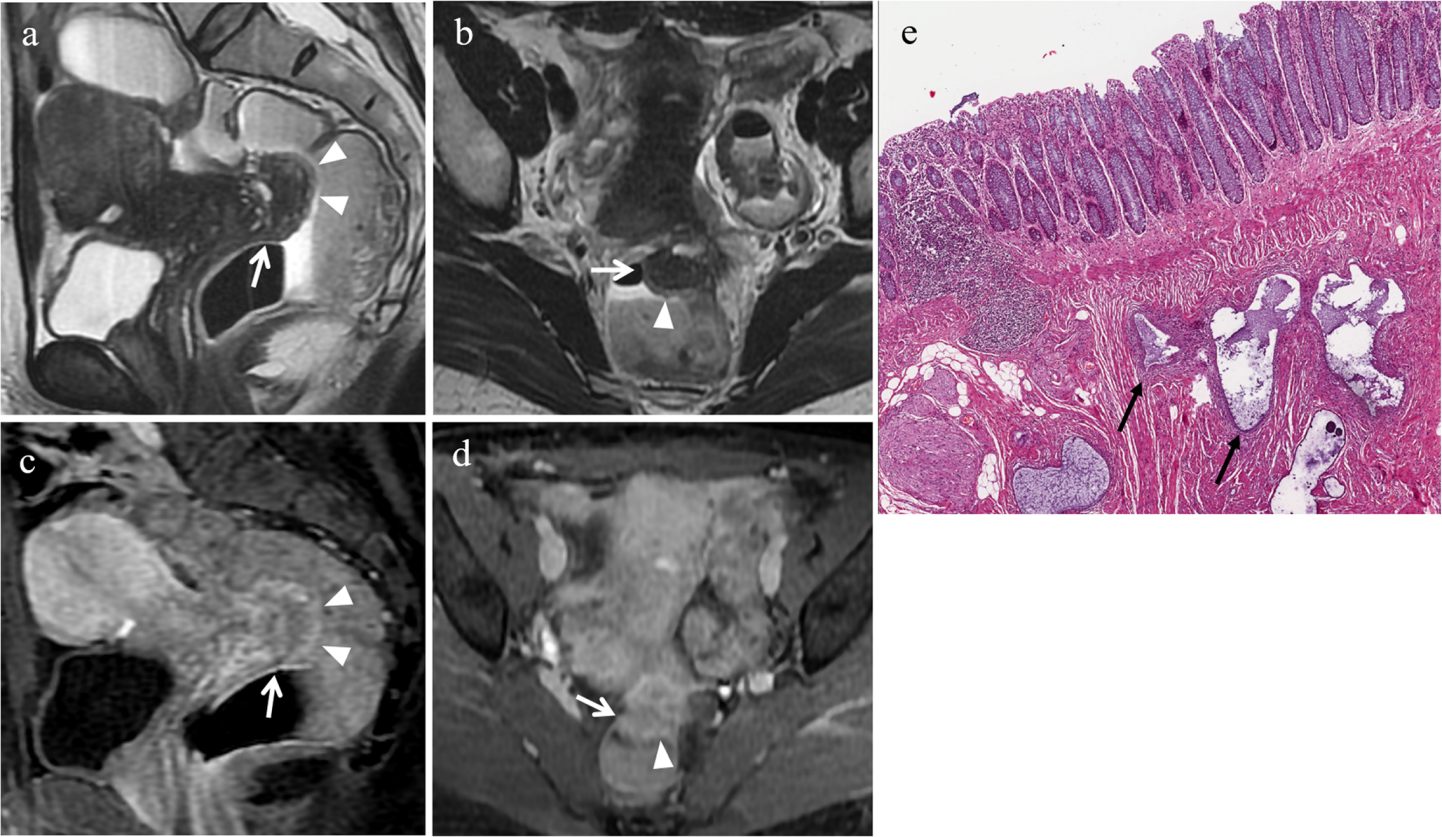
Endometriosis of the anterior rectal wall in a 48-year-old woman with dysmenorrhea, chronic pelvic pain and catamenial diarrhoea. **(a)** Sagittal and **(b)** oblique coronal T2-weighted images, and **(c)** sagittal and **(d)** oblique coronal contrast-enhanced fat-suppressed T1-weighted images show an endometriotic nodule (white arrows) infiltrating the muscular layer of the anterior rectal wall. The lesion has a “fan shaped” configuration with the base adhering to the rectal wall and the apex oriented toward the retrocervical region. The implant demonstrates isointense signal compared to muscle on T2-weighted and T1-weighted sequences; the slightly high signal at the luminal side (white arrowheads in a and b) corresponds to (sub)mucosal thickening and enhances after intravenous administration of contrast material (white arrowheads in c and d). **(e)** Photomicrograph (H&E 40X). Endometriosis in the muscularis propria (black arrows)

**Fig. 21:**
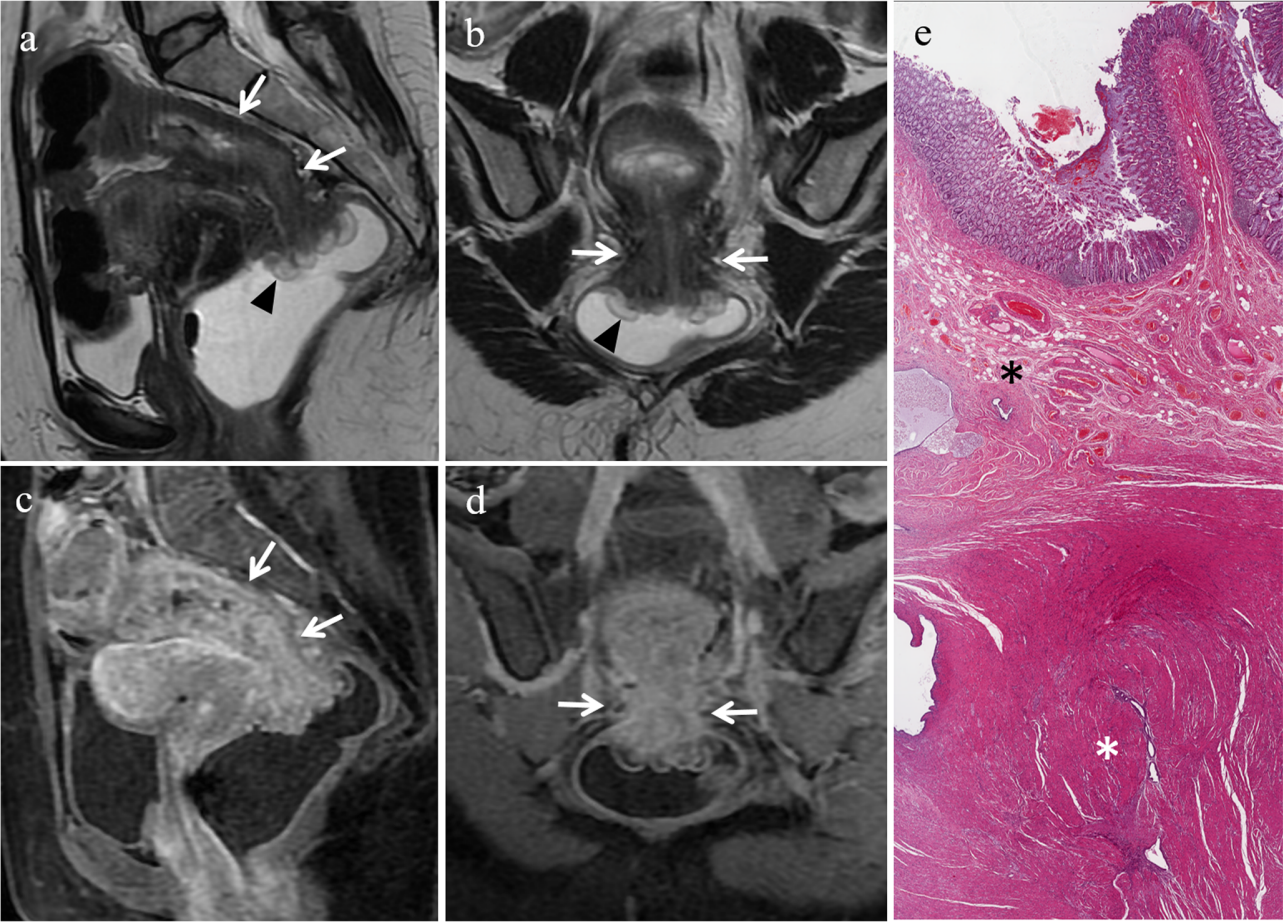
Endometriosis of the rectal wall in a 45-year-old woman with cyclic hematochezia, constipation, pencil-like stools and episodes of intestinal subocclusion, who underwent previous right ureteral stenting. **(a)** Sagittal and **(b)** oblique coronal T2-weighted images, and **(c)** sagittal and **(d)** oblique coronal contrast-enhanced fat-suppressed T1-weighted images show an endometriotic lesion (white arrows) infiltrating the muscular and submucosal layers of the rectal wall. The lesion extends longitudinally for about 7 cm and determines severe stenosis. Note the hyperintense signal of the (sub)mucosal layer protruding into the rectal lumen (black arrowheads in a and b). **(e)** Photomicrograph (H&E 25X). Endometriosis in submucosa (black *) and in muscularis propria (white *)

Anatomically the rectosigmoid wall is characterized by four intraperitoneal layers: serosa, outer longitudinal muscularis, inner circular muscularis and mucosa [51]. The implants generally involve the serosal surface but may invade the underlying muscular and submucosal layers; only rarely implants erode the mucosa causing cyclic rectal bleeding. Typically, endometriotic lesions infiltrating the anterior rectal wall have a characteristic “fan shaped” configuration (or a pyramidal shape, with the base adhering to the rectal wall and the apex oriented anteriorly toward the retrocervical region). The core of the lesion shows isointense signal compared to muscle on T2-weighted and T1-weighted sequences and at histopathology corresponds to thickening and distortion of the muscularis propria and smooth muscle hyperplasia. The overlying layer, hyperintense on T2-weighted images, at the luminal side of the bowel wall corresponds to (sub)mucosal thickening, as a consequence of “non-specific inflammation” with or without infiltration of endometriosis (Figs. 20 and 21) [17]. When the longitudinal extent of the parietal lesion along the bowel wall is short, a pattern of intraluminal endophytic growth, called “mushroom cap”, may be observed [52].

In a recently published meta-analysis the sensitivity and specificity of MRI for the diagnosis of rectosigmoid colon endometriosis were 83% and 88%, respectively [46].

MR imaging is useful to predict infiltration of the muscular layer of the bowel with a sensitivity of 100% and specificity of 75%. On the other hand, it is of limited value in diagnosing (sub)mucosal infiltration, as (sub)mucosal thickening may be caused by edema without infiltration of endometriosis. Nevertheless, extensive irregularities of the (sub)mucosal layer may raise suspicion of (sub)mucosal involvement [17]. Transvaginal US after bowel preparation is the best imaging modality for determining which bowel wall layers are affected [12].

Adhesions, strictures, and bowel obstruction may occur representing complications of intestinal endometriosis.

The surgical procedure depends on the lesion size (>2 cm or 3 cm), degree of infiltration (muscularis invasion), percentage of circumference involvement, number and location of intestinal lesions [3, 17, 51, 53]. The distance between the inferior margin of the nodule and the anal border in another important information for surgical planning and can be easily estimated at MRI on sagittal T2-weighted sequences [3].

The multilayer structure of the rectosigmoid wall lead to several possibilities of intestinal resection depending on the degree of invasion (Table 3) [51]. Regarding clinical symptoms, segmental bowel resection is performed when patients do not respond to hormonal therapy and/or there is suspicion of a clinically relevant stenosis of the bowel [17].

**Table 3:** Different laparoscopic bowel resection techniques depending on the degree of invasion [51]

Laparoscopic technique	Description	Indication
Laparoscopic rectosigmoid shaving resection	Removal of the lesion only, followed by primary suture	Lesions <1 cm
Laparoscopic “mucosal skinning”	Removal of both muscular layers followed by interrupted sutures	Plaques located on the anterior rectosigmoid wall
Laparoscopic rectosigmoid discoid resection	Full thickness resection of the anterior bowel wall followed by two-layer suture	Single lesions <3 cm involving less than one third of the circumference
Laparoscopic rectosigmoid segmental resection	Bowel resection followed by end-to-end anastomosis with sparing of mesenteric nerves	Multiple or bigger lesions determining bowel distortion

Differential diagnosis includes rectal cancer and metastatic implants to the bowel.

Rectal endometriosis may be difficult to differentiate from colorectal carcinoma when presenting with non-specific clinical and imaging features or in cases of incidental bowel wall thickening on MR imaging [54]. In this regard it is important to remember that endometriosis is an extrinsic lesion which starts at the serosa, infiltrates the muscular layer and only rarely invades the mucosa [3], whereas colorectal carcinoma is an intrinsic lesion starting at the mucosa. Busard et al. [54] have proposed qualitative assessment of high b-value diffusion-weighted images as a valuable, non-invasive tool to facilitate differentiation between endometriosis infiltrating the bowel and colorectal carcinoma. Both colorectal carcinoma and endometriosis infiltrating the bowel demonstrate low ADC values; nevertheless, colorectal carcinoma shows high signal intensity on DWI images due to high cellularity, whereas endometriosis displays hypointense signal intensity on DWI images. The low signal intensity of endometriosis infiltrating the bowel on DW images could be due to the “T2-blackout effect” of these lesions, that are very hypointense on T2-weighted imaging because of smooth muscle hyperplasia and fibrous tissue; their restricted diffusion (low ADC) might be explained by low water content and fibres blocking diffusion.

## Unusual locations

An estimated 12% of patients with pelvic endometriosis have extragenital/extrapelvic endometriosis [20]. This clinical condition is often associated with considerable diagnostic delay and morbidity. Among atypical locations, diaphragmatic and sciatic nerve endometriosis are probably the most relevant for both clinical and therapeutic implications.

### Diaphragmatic endometriosis

Diaphragmatic endometriosis is thought to be rare, accounting for about 1.5% in gynaecological surgical series [19]. It is symptomatic in up to 47% of cases [18], nevertheless it is also largely under-diagnosed resulting in delayed diagnosis of up to 10 years from the onset of symptoms [19]. Association with pelvic endometriosis is almost constant, thus confirming the Sampson’s transplantation theory of retrograde menstruation through the fallopian tubes as an etiological mechanism and the endometrial cells clockwise peritoneal circulation through the right paracolic gutter toward the ipsilateral subdiaphragmatic region.

**Fig. 22:**
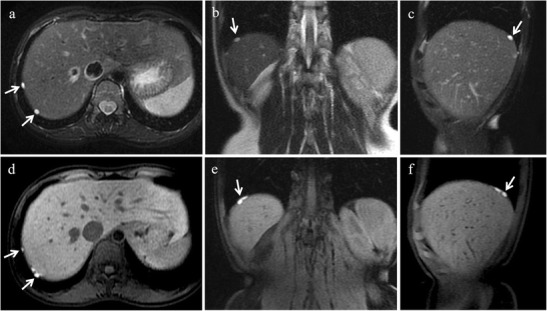
Diaphragmatic endometriosis in a 28-year-old woman with right-sided basithoracic chest pain associated with menses, who underwent a previous surgical intervention for endometrioma of the left ovary. **(a)** Axial, **(b)** coronal, **(c)** sagittal T2-weighted images, and **(d)** axial, **(e)** coronal and **(f)** sagittal fat-suppressed T1-weighted images show right-sided hyperintense nodular diaphragmatic implants (white arrows). The lesions are better depicted on fat-suppressed T1-weighted sequences

MRI allows the diagnosis of diaphragmatic endometriotic implants with a sensitivity of 78–83% [19]. Lesions are right-sided and predominantly posterior. Their structure is non-purely cystic; the morphological appearance can be of nodule, micronodule clustering (< 5 mm) or plaque lesion. Focal liver herniation, corresponding to diaphragmatic defects, may also occur [19]. Typically, diaphragmatic implants are hyperintense on both fat-suppressed T1-weighted sequences (consistent with blood products) and T2-weighted sequences, and are better depicted on fat-suppressed T1-weighted sequences [19] (Fig. 22).

However, MRI may fail to detect small superficial diaphragmatic nodules; hence a negative MR examination does not exclude the diagnosis of diaphragmatic endometriosis in women with suggestive symptoms and would not eliminate the need to surgically investigate the upper abdomen and diaphragm [18].

Suppressive hormonal treatment can lead to resolution or significant relief of symptoms. Thoracic surgery is usually only performed in cases of recurrent catamenial pneumothorax [19].

### Endometriosis of the sciatic nerve

Regarding peripheral nervous system endometriosis, the most frequently involved site is the sacral plexus (57%), followed by the sciatic nerve (39%) [55]. Extrapelvic sciatic endometriosis is due to implantation of endometrial tissue in the sciatic nerve, usually in the region of the sciatic notch [2].

**Fig. 23:**
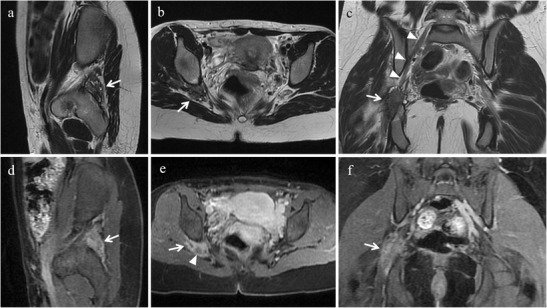
Extrapelvic sciatic nerve endometriosis in a 31-year-old woman who has been suffering from cyclic sciatica for about 2 years. **(a)** Sagittal, **(b)** axial and **(c)** coronal T2-weighted images show hypointense spiculated soft-tissue thickening centred around the right sciatic nerve at the sciatic notch (white arrows). **(d)** Sagittal, **(e)** axial and **(f)** coronal contrast-enhanced fat-suppressed T1-weighted images display enhancement of the mass (white arrows). Note the sciatic nerve cephalad to the lesion (white arrowheads in c) and within the lesion (white arrowhead in e)

**Fig. 24:**
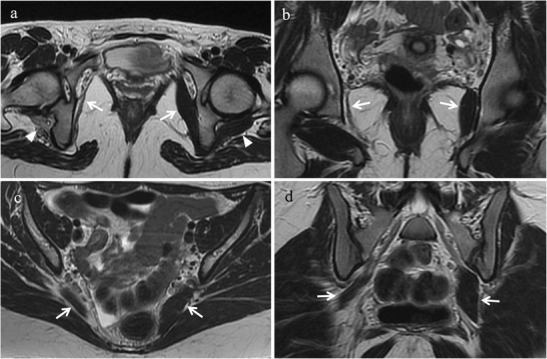
Indirect MR findings of sciatic nerve endometriosis. The same patient as in Fig. 23. **(a)** Axial and **(b)** coronal T2-weighted images show atrophy of right obturator internus (white arrows) and gemellus superior muscle (white arrowhead in a) compared with the contralateral ones (white arrows and white arrowhead in a). **(c)** Axial and **(d)** coronal T2-weighted images display atrophy of right piriformis muscle compared with the contralateral one (white arrows)

MRI is the diagnostic method of choice to demonstrate endometriosis lesions along nerve pathways and to precisely identify the site of the lesion [56]. The appearance of neurotropic endometriotic lesions can be variable as solid or complex cystic masses with thick or thin walls [21]. At MR imaging they usually manifest as hypointense irregular spiculated soft-tissue thickening centred around the sciatic nerve at the sciatic notch on T2-weighted images; small intermingled hyperintense foci, indicating bloody content, may frequently be observed on fat-suppressed T1-weighted images [2] (Fig. 23). Indirect MR findings may also be found as atrophy in corresponding target muscles of the sciatic nerve and the lumbosacral plexus (gluteal muscles, obturator internus muscle, quadratus femoris muscle) [22] (Fig. 24).

Electromyography can demonstrate signs of denervation and slowing of conduction speed. It can be useful to differentiate between root and peripheral nerve involvement [21].

This clinical condition is often associated with considerable diagnostic delay and morbidity. Since the prognosis depends on the interval between the onset of symptoms and diagnosis, early diagnosis and treatment are important in order to prevent irreversible damage to the sciatic nerve [21]. Therapeutic options include pharmacologic treatment and surgical therapy. The differential diagnosis includes benign neurogenic tumours [21].

## Conclusions

Endometriosis is a chronic condition affecting women during the reproductive lifespan. Diagnosis of endometriosis must take into account clinical symptoms, physical examination, laboratory tests and different imaging techniques. Since pelvic anatomy is complex and may vary with distortion by invasive endometriosis, the radiologist must be aware of both normal and deranged anatomy.

The ideal purpose of surgery is a therapeutic and effective intervention based on a careful preoperative evaluation. From this point of view, the role of MR imaging to help diagnose and plan surgical strategy is critical in the management of the disease. Preoperative detection of all endometriotic lesions is recommended to choose the surgical approach and to plan a multidisciplinary team work [29]. This multidisciplinary approach including radiologists, gynaecologists, urologists, gastrointestinal surgeons, and (in selected cases) neurosurgeons, is recommended to improve diagnostic imaging accuracy and patients’ outcome, and to reduce postoperative complication rates. The recent awareness that endometriosis may be medically treated based on strong clinical suspicion [57] and that laparoscopy should be intended for surgical treatment, not for diagnostic purposes [58], furtherly enhance the role of non-invasive diagnostic procedures and particularly of MR imaging.

In any case, due to the complexity of the disease, it is appropriate to centralize the overall care of endometriosis patients to reference centres in order to pursue a patient-centred approach tailored to the patient’s specific condition and desires.
